# Direct Bilirubin Levels Predict Long-Term Outcomes in Patients With Acute Coronary Syndrome Under Different Glucose Metabolism Status: A 6.5-Year Cohort Study of Three-Vessel Disease

**DOI:** 10.3389/fcvm.2021.715539

**Published:** 2021-08-12

**Authors:** Yue Liu, Ce Zhang, Lin Jiang, Jian Tian, Xue-yan Zhao, Jing-jing Xu, Ru Liu, Bo Xu, Ru-tai Hui, Run-lin Gao, Jin-qing Yuan, Lian-jun Xu, Lei Song

**Affiliations:** ^1^Department of Cardiology, Fuwai Hospital, Chinese Academy of Medical Science and Peking Union Medical College, Beijing, China; ^2^Catheterization Laboratories, Fuwai Hospital, National Center for Cardiovascular Diseases, Chinese Academy of Medical Sciences and Peking Union Medical College, Beijing, China

**Keywords:** acute coronary syndrome, 3-vessel disease, bilirubin, prognosis, SYNTAX score II

## Abstract

**Background:** There is controversy over the relationship between bilirubin and coronary artery disease. This study aimed to evaluate the predictive value of direct bilirubin (DB) in patients with complex acute coronary syndrome (ACS).

**Methods:** From April 2004 to February 2011, 5,322 ACS patients presenting with three-vessel disease were consecutively enrolled. Disease severity and complexity were determined by SYNTAX score (SS) and SS II. The primary endpoint was all-cause death, and the secondary endpoints were major adverse cardiovascular and cerebrovascular events (MACCE). Stratification of normal glucose regulation, prediabetes, and diabetes was based on a previous diagnosis, hypoglycemic medications, fasting blood glucose, and hemoglobin A1c.

**Results:** Subjects were divided into quartiles according to baseline DB (μmol/L): Q1 (0–1.6), Q2 (1.61–2.20), Q3 (2.21–2.80), and Q4 (>2.80). Multivariable logistic regression analysis showed that DB was an independent predictor of intermediate–high SS. During a median follow-up time of 6.5 years, elevated DB was associated with more all-cause death (*p* < 0.001) but not MACCE. DB remained to be predictive of all-cause death in the multivariable Cox regression model (Q2 vs. Q1: HR 1.043, 95% CI 0.829–1.312, *p* = 0.719; Q3 vs. Q1: HR 1.248, 95% CI 1.001–1.155, *p* = 0.048; Q4 vs. Q1: HR 1.312, 95% CI 1.063–1.620, *p* = 0.011). When subjects are stratified according to glucose metabolism regulation and treatment strategies, the predictivity of DB was only profound in patients with diabetes or with conservative treatment. Additionally, incorporating DB further improved the discrimination and reclassification abilities of SS II for risk prediction.

**Conclusion:** DB is a potential biomarker for predicting lesion severity and long-term outcomes in ACS patients.

## Introduction

Ischemic heart disease as well as stroke remains to be the leading cause of death worldwide (1, 2). Acute coronary syndrome (ACS), including ST-segment elevated myocardial infarction (STEMI), non-STEMI (NSTEMI), and unstable angina pectoris, is the focus area because of its volatile nature and adverse prognosis (3, 4). Despite advances in medicine and invasive approaches, patients with complex ACS, i.e., three-vessel disease (3VD), still suffer from life-threatening disease. Unconventional biomarkers have been increasingly investigated for better management of this high-risk population (5–7).

Bilirubin, the end product of heme metabolism, is an endogenous antioxidant (8). There are cumulative studies that support the protective effect of total bilirubin (TB) against the onset of cardiovascular disease (9–11). Nevertheless, a few contradictory findings in established ACS make this issue inconclusive (12–14). In light of direct bilirubin (DB, also known as conjugated bilirubin), scarce evidence is available regarding its effect in either diagnosis or risk stratification of ACS.

Oxidative stress exerts a great impact on insulin resistance, which raises a question on whether bilirubin plays a role in this pathway (15). A study in obese patients with type 2 diabetes found that bilirubin acted as a protective factor in insulin resistance (OR = 0.744, 95% CI: 0.590–0.938, *p* = 0.012) (16). Nevertheless, an interesting result was also observed in that TB, DB, and indirect bilirubin levels were higher in the diabetes group than in the normal metabolism population. Another research also showed a positive relationship between DB and incident type 2 diabetes, while no significance in either TB or indirect bilirubin (17). However, there was no literature discussing bilirubin, especially DB, in diabetic patients with ACS.

Therefore, this study aims to evaluate whether the level of DB at admission can indicate angiographic manifestations and long-term prognosis of ACS patients with all three-vessel stenoses.

## Materials and Methods

### Study Population

We performed an observational, prospective, single-center study in Fuwai Hospital, Chinese Academy of Medical Sciences. A total of 8,943 consecutive patients with 3VD (defined as angiographic stenosis of ≥50% in all three main coronary arteries including left anterior descending, circumflex, and right coronary artery, with or without the left main artery involved) were enrolled from April 2004 to February 2011 (7). The treatment strategy [medical therapy alone, percutaneous coronary intervention (PCI), or coronary artery bypass grafting] was made based on contemporary practice guidelines, heart team judgement, and patients' preferences.

The following participants were excluded sequentially: (1) those with presentation of stable coronary artery disease; (2) those with abnormal liver function (defined as a serum aspartate aminotransferase or alanine aminotransferase >100 U/L and a serum c-glutamyl transferase >100 U/L); and (3) those lacking records of bilirubin. Patients were stratified into quartiles according to DB on admission: Q1 (0–1.60 μmol/L), Q2 (1.61–2.20 μmol/L), Q3 (2.21–2.80 μmol/L), and Q4 (>2.80 μmol/L).

Diabetes was defined as newly diagnosed diabetes [fasting blood glucose ≥7.0 mmol/L or hemoglobin A1c (HbA1c) ≥6.5%] or known diabetes. Patients with prediabetes were those without established diabetes but with fasting glucose ranging from 5.6 to 7.0 mmol/L and/or HbA1c ranging from 5.7 to <6.5%.

The Review Board of Fuwai Hospital approved the study protocol in accordance with the Declaration of Helsinki, and all patients provided written informed consent.

### Laboratory and Angiographic Evaluations

Blood samples were obtained by venipuncture at admission after fasting overnight. TB and DB (measured by Hitachi 7060 automatic biochemical analysis), together with blood cells, fasting blood glucose, lipid profiles, and other biomarkers, were measured using standard laboratory methods. Experienced cardiologists who were blinded to the clinical data assessed SYNTAX score (SS) and SS II. The algorithms were described in detail in previous studies and were available online (http://www.syntaxscore.com) (18, 19).

### Outcomes

Patients were followed up by telephone, letter, or clinic visit. The last follow-up was finished in 2016, with a response rate of 80.6%. All events were carefully checked and verified by an independent group of clinical physicians. Investigator training, blinded questionnaire filling, and telephone recording were performed to obtain high-quality data. The primary endpoint was all-cause death. Secondary endpoints included cardiac death and major adverse cardiovascular and cerebrovascular events (MACCE), a composite of all-cause death, myocardial infarction (MI), unplanned revascularization, and/or stroke. All deaths were considered cardiac unless an undisputed non-cardiac cause was present.

### Statistical Analysis

Data are expressed as mean ± standard deviation, medians (interquartile range), or numbers (%). Differences in baseline characteristics and outcomes among quartiles were evaluated by one-way analysis of variance (ANOVA) for numerical data and the chi-squared test for categorical data. Spearman's correlation was used to assess the associations between DB and other parameters when one or both of them were not normally distributed. Determine the independent predictors for intermediate-high SS by means of multivariable logistic regression analysis. Survival analyses were presented by Kaplan-Meier curves and Cox proportional regression models. Establish different models to judge the independence of DB. Covariates in Model 1 were age, gender, and body mass index. The covariate in Model 2 was SS II. And in Model 3, there were SS II, glucose metabolism, treatment strategy, previous history of stroke, body mass index, low-density lipoprotein, blood pressure, high-sensitivity C-reactive protein, and liver enzymes. Additionally, we used the C-index, continuous net reclassification improvement (NRI), and integrated discrimination improvement (IDI) to determine whether DB added incremental predictive value of SS II. A two-tailed *p* value of < 0.05 was considered to be statistically significant. All of the analyses were performed with the SPSS Statistics version 20.0 (SPSS Inc., Chicago, Illinois, USA) and R software version 3.4.3 (R Core Team, Vienna, Austria).

## Results

**Figure 1:**
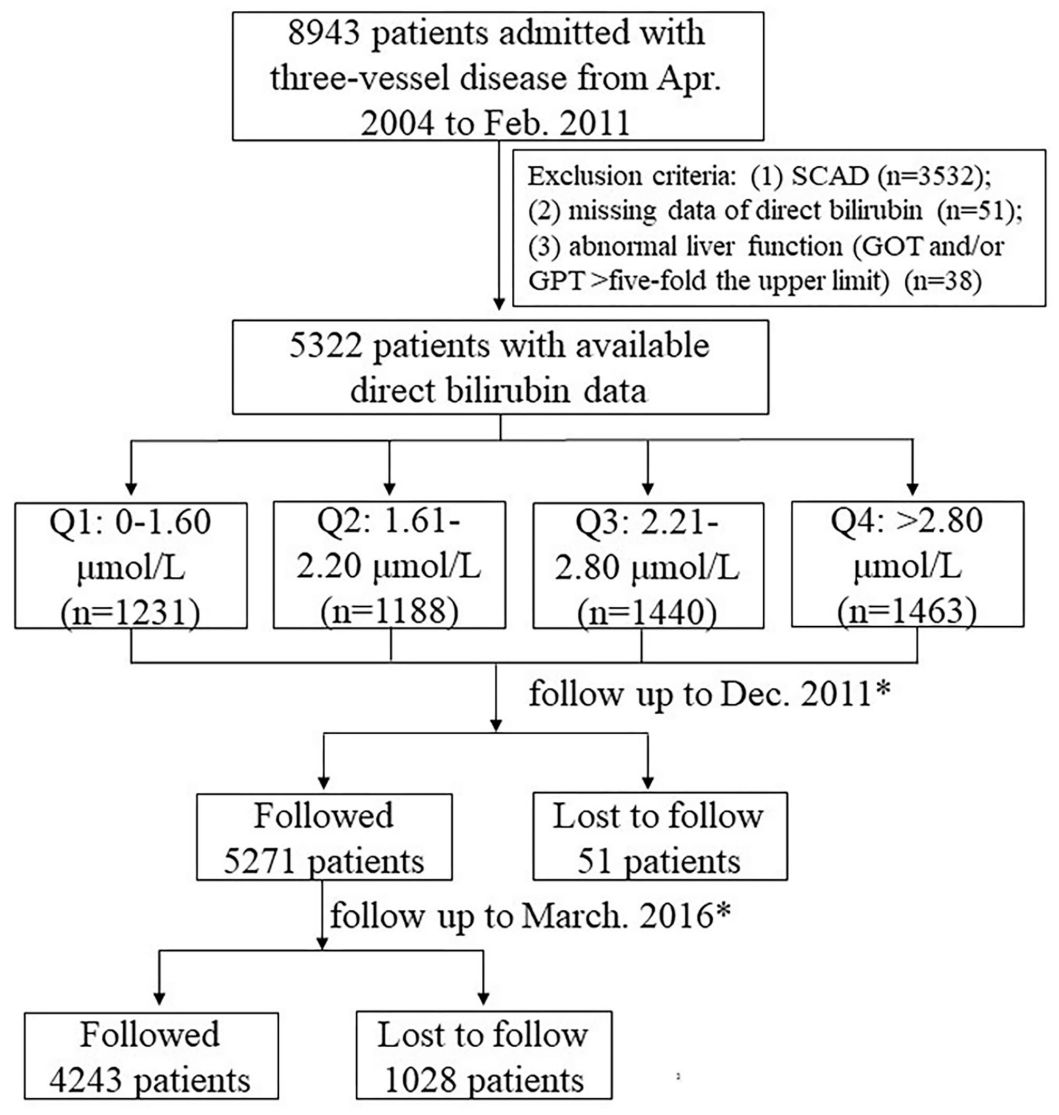
Flowchart of patient enrollment.

The flowchart of patient selection is depicted in Figure 1. Of 5,322 available cases, the average age was 61.3 years, and the majority were male (78.6%). A total of 45.4% of patients had received PCI, and 26.6% had undergone grafting surgery. Diabetic patients accounted for 41.9% (2,227/5,322), prediabetes accounted for 23.1% (1,231/5,322), and 35% (1,864/5,322) were under normal glucose regulation.

Baseline characteristics across DB quartiles are shown in Tables 1, 2. Patients with higher DB were older, more often to be male, and had higher proportions of previous history of MI and smoking. However, fewer cases of diabetes or hyperlipidemia were found following DB elevation.

**Table 1:** Baseline characteristics across DB quartiles.

	**Overall**	**Q1**	**Q2**	**Q3**	**Q4**	***p*-value**
	**(*n* = 5,322)**	**(*n* = 1,231)**	**(*n* = 1,188)**	**(*n* = 1,440)**	**(*n* = 1,463)**	
Age, years	61.3 ± 10.2	60.8 ± 10.3	61.1 ± 10.1	61.3 ± 10.1	62.1 ± 10.2	0.006
Female gender	1,138 (21.4)	407 (33.1)	273 (23.0)	280 (19.4)	178 (12.2)	<0.001
BMI, kg/m^2^	25.7 ± 3.0	25.6 ± 3.1	25.7 ± 3.0	25.9 ± 3.0	25.8 ± 3.1	0.036
Diabetes	2,227 (41.8)	565 (45.9)	479 (40.3)	559 (38.8)	624 (42.7)	<0.001
Hypertension	3,638 (68.4)	841 (68.3)	835 (70.3)	959 (66.6)	1,003 (68.6)	0.247
Dyslipidemia	2,990 (56.2)	724 (58.8)	717 (60.4)	773 (53.7)	776 (53.0)	<0.001
Previous MI	1,729 (32.5)	342 (27.8)	376 (31.6)	485 (33.7)	526 (38.0)	<0.001
Previous stroke	551 (10.4)	130 (10.6)	108 (9.1)	146 (10.1)	167 (11.4)	0.268
Peripheral artery disease	402 (7.6)	86 (7.0)	108 (9.1)	101 (7.0)	107 (7.3)	0.150
COPD	66 (1.2)	6 (0.5)	17 (1.4)	22 (1.5)	21 (1.4)	0.058
Smoking, *n* (%)	2,960 (55.6)	602 (48.9)	678 (57.1)	827 (57.4)	853 (58.3)	<0.001
Clinical presentation						<0.001
STEMI	1,073 (20.2)	258 (21.0)	209 (17.6)	257 (17.8)	349 (23.9)	
NSTE-ACS	4,249 (79.8)	973 (79.0)	979 (82.4)	1,183 (82.2)	1,114 (76.1)	
TB, μmol/L	11.4–17.8	11.4 ± 4.0	13.4 ± 5.4	15.3 ± 7.6	21.0 ± 12.5	<0.001
Fasting glucose, mmol/L	6.24 ± 2.24	6.36 ± 2.51	6.08 ± 1.99	6.14 ± 2.09	6.38 ± 2.33	<0.001
hsCRP, mg/L	1.09–6.75	1.10–5.14	1.02–5.50	1.07–6.50	1.20–10.05	0.037
Glycated hemoglobin, %	6.56 ± 1.48	6.76 ± 1.50	6.47 ± 1.42	6.52 ± 1.37	6.50 ± 1.57	0.001
LDL, mmol/L	2.53 ± 0.82	2.70 ± 0.91	2.58 ± 0.86	2.52 ± 0.70	2.36 ± 0.77	<0.001
HDL, mmol/L	1.04 ± 0.26	1.05 ± 0.26	1.04 ± 0.26	1.05 ± 0.25	1.02 ± 0.26	0.009
Total cholesterol, mmol/L	4.57 ± 1.06	4.88 ± 1.17	4.61 ± 1.07	4.56 ± 0.90	4.39 ± 1.01	<0.001
NT-proBNP	450–1,044	429–949	454–979	441–1,044	477–1,179	<0.001
GFR, ml/min·1.73 m^2^	82 ± 18	83 ± 19	83 ± 19	82 ± 17	82 ± 18	0.001
LVEF <40%	374 (7.0)	55 (4.5)	63 (5.3)	98 (6.8)	158 (10.8)	<0.001

*Variables were summarized as mean ± standard deviation (SD), median (interquartiles), or n (%).*

*BMI, body mass index; COPD, chronic obstructive pulmonary disease; GFR, glomerular filtration rate; HDL, high-density lipoprotein; hsCRP, high-sensitivity C-reactive protein; LDL, low-density lipoprotein; LVEF, left ventricular ejection fraction; MI, myocardial infarction; NSTE-ACS, non-ST-elevated ACS; NT-proBNP, N-terminal pro-B-type natriuretic peptide; STEMI, ST-elevated myocardial infarction*.

**Table 2:** Angiographic characteristics and treatment across DB quartiles.

	**Overall**	**Q1**	**Q2**	**Q3**	**Q4**	***p*-value**
	**(*n* = 5,322)**	**(*n* = 1,231)**	**(*n* = 1,188)**	**(*n* = 1,440)**	**(*n* = 1,463)**	
Treatment strategy						0.483
PCI	2,416 (45.4)	572 (46.5)	553 (46.5)	655 (45.5)	636 (43.5)	
CABG	1,415 (26.6)	331 (26.9)	318 (26.8)	376 (26.1)	390 (26.7)	
Medical therapy	1,491 (28.0)	328 (26.6)	317 (26.7)	409 (28.4)	437 (29.9)	
SS	25.6 ± 11.0	24.9 ± 9.5	25.7 ± 9.8	25.6 ± 10.8	26.2 ± 13.0	0.024
**Medicine during hospitalization**
Aspirin	5,087 (95.6)	1,184 (96.2)	1,135 (95.5)	1,376 (95.6)	1,392 (95.1)	0.634
Clopidogrel	3,003 (56.4)	716 (58.2)	681 (57.3)	785 (54.5)	821 (56.1)	0.251
β-Blocker	4,672 (87.8)	1,090 (88.5)	1,041 (87.6)	1,261 (87.6)	1,280 (87.5)	0.832
CCB	1,926 (36.2)	463 (37.6)	460 (38.7)	515 (35.8)	488 (33.4)	0.023
Statin	3,692 (69.4)	853 (69.3)	839 (70.6)	1,002 (69.6)	998 (68.2)	0.609
RAS inhibitors	2,897 (54.4)	679 (55.2)	647 (54.5)	764 (53.1)	807 (55.2)	0.642

*Variables were summarized as mean ± standard deviation (SD) or n (%).*

*CABG, coronary artery bypass grafting; CCB, calcium channel blocker; RAAS, renin-angiotensin system; SYNTAX, Synergy between PCI with TAXUS and Cardiac Surgery*.

Spearman correlation analyses demonstrated statistically significant but weak correlations between DB and N-terminal pro-B-type natriuretic peptide (NT-proBNP) (0.109, < 0.001), high-sensitivity C-reactive protein (0.097, < 0.001), age (0.051, <0.001), SS (0.044, 0.002), low-density lipoprotein (−0.142, < 0.001), and glycated hemoglobin (−0.081, < 0.001).

In agreement with the correlation analysis, DB (as a categorical variable) as well as age, peripheral vessel disease, and glycated hemoglobin was an independent predictor of intermediate–high SS (>22) in a multivariable logistic regression model (Table 3).

**Table 3:** Multivariate logistic analysis for intermediate–high SS.

	**OR**	**95% CI**		***p*-value**
DB (vs. Q1)			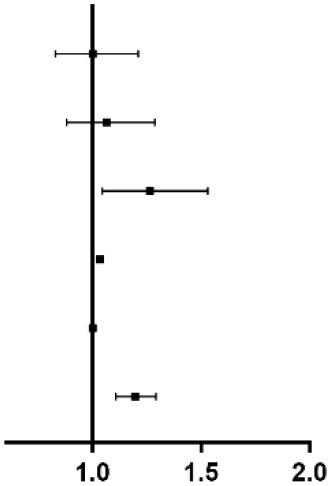	0.046
Q2	1.001	0.828–1.210		0.995
Q3	1.064	0.881–1.286		0.519
Q4	1.264	1.044–1.530		0.016
Age	1.033	1.020–1.047		0.001
NT-proBNP	1.000	1.000–1.000		0.001
Glucose metabolism status	1.196	1.107–1.292		<0.001

*CI, confidence interval; OR, odds ratio; SYNTAX, Synergy between PCI with TAXUS and Cardiac Surgery*.

The median follow-up period was 6.5 (5.0–8.6) years. All patients had completed at least one follow-up. The last follow-up finished in March 2016 with a response rate of 79.7%. In total, 901 (16.9%) patients died, of which 471 (8.9%) were due to cardiac cause, and 1,777 (33.4%) had MACCE.

**Figure 2:**
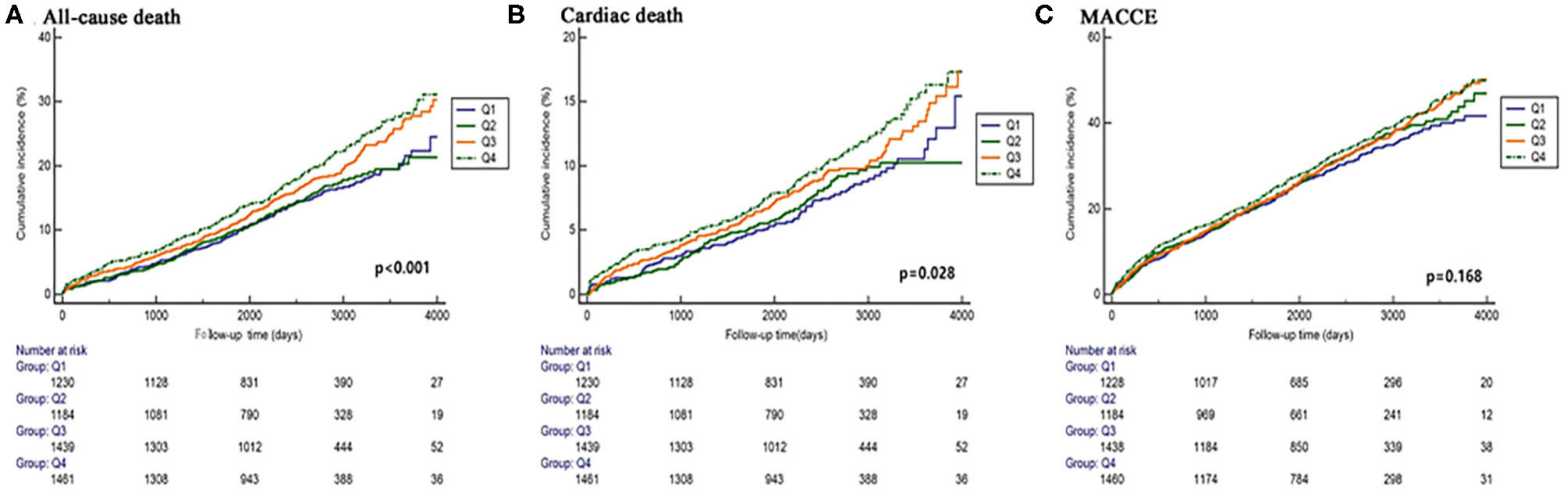
Kaplan–Meier curves of the primary and secondary endpoints across DB quartiles (**A**–**C**). Cumulation incidence curves for all-cause death (**A**), cardiac death (**B**), and MACCE (**C**). MACCE, major adverse cardiac and cerebrovascular events.

**Figure 3:**
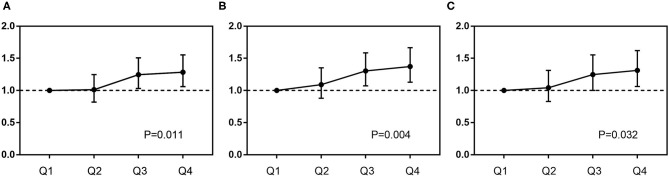
Predictive value of DB for mortality in different Cox proportional models. Covariates in Model 1 **(A)**: age, sex, and body mass index; Model 2 **(B)**: SS II; Model 3 **(C)**: SS II, glucose metabolism status, treatment strategies, previous history of stroke, body mass index, blood pressure, low-density lipoprotein-cholesterol, high-sensitivity C-reactive protein, and liver enzymes.

Elevated DB was significantly associated with more all-cause death [14.7 vs. 14.4 vs. 18.5 vs. 19.3%, *p* < 0.001] and cardiac death (7.6 vs. 7.5 vs. 9.7 vs. 10.2%, *p* = 0.025), while no difference was found in any other adverse events (Figure 2 and Table 4). Regardless of different adjusting models, DB remained to be an independent predictor of all-cause death in multivariable Cox analysis (in Model 3, Q2 vs. Q1: HR 1.043, 95% CI 0.829–1.312, *p* = 0.719; Q3 vs. Q1: HR 1.248, 95% CI 1.001–1.155, *p* = 0.048; Q4 vs. Q1: HR 1.312, 95% CI 1.063–1.620, *p* = 0.011) (Figure 3).

**Table 4:** Clinical outcomes across bilirubin quartiles.

	**Overall**	**Q1**	**Q2**	**Q3**	**Q4**	***p*-value**
	**(*n* = 5,322)**	**(*n* = 1,231)**	**(*n* = 1,188)**	**(*n* = 1,440)**	**(*n* = 1,463)**	
Death	901 (16.9)	181 (14.7)	171 (14.4)	266 (18.5)	283 (19.3)	<0.001
Cardiac death	471 (8.9)	94 (7.6)	89 (7.5)	139 (9.7)	149 (10.2)	0.025
MI	315 (5.9)	83 (6.7)	66 (5.6)	87 (6.0)	79 (5.4)	0.470
Revascularization	462 (8.7)	111 (9.0)	115 (9.7)	116 (8.1)	120 (8.2)	0.423
Stroke	395 (7.4)	93 (7.6)	93 (7.8)	102 (7.1)	107 (7.3)	0.900
MACCE	1,777 (33.4)	385 (31.3)	382 (32.2)	502 (34.9)	508 (34.7)	0.119

*MACCE, major adverse cardiovascular and cerebrovascular events; MI, myocardial infarction*.

**Figure 4:**
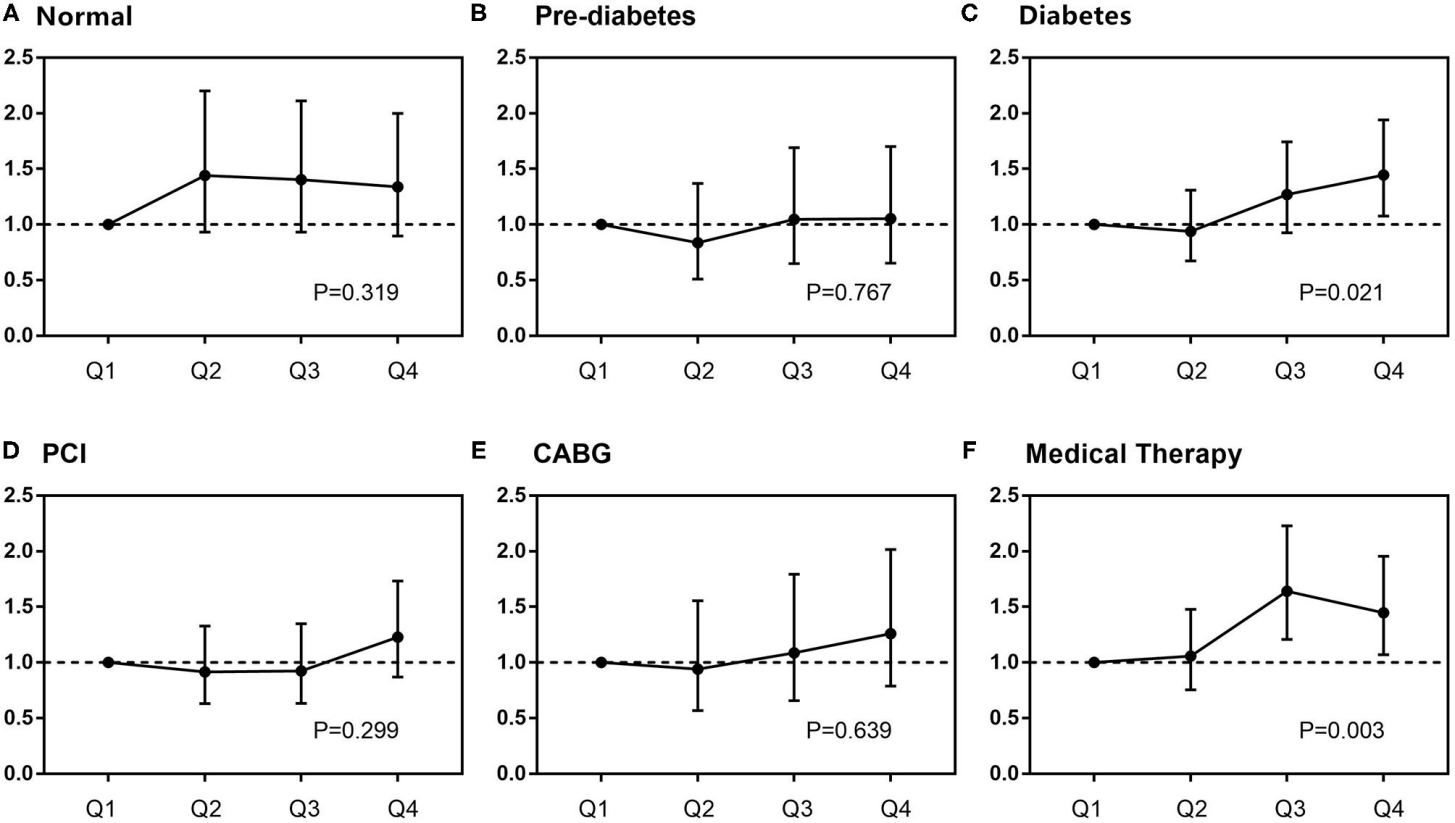
Subgroup analysis of the relationship between DB and mortality. In different glucose metabolism statuses **(A–C)**, adjusted variables were SS II, treatment strategies, previous history of stroke, body mass index, blood pressure, low-density lipoprotein-cholesterol, high-sensitivity C-reactive protein, and liver enzymes. In different treatments **(D–F)**, adjusted variables were SS II, glucose metabolism status, previous history of stroke, body mass index, blood pressure, low-density lipoprotein-cholesterol, high-sensitivity C-reactive protein, and liver enzymes. CABG, coronary artery bypass grafting; PCI, percutaneous coronary intervention.

Subgroup analysis further showed that DB maintained its independent predictivity for long-term mortality only in the diabetic subjects, whereas it did not reach any statistical significance in either pre-diabetes or normal glucose regulation ones (Table 5 and Figure 4). A similar result was also found in those receiving medical therapy alone (Figure 4).

**Table 5:** Predictive performance of DB under different conditions of glucose metabolism regulation.

	Model 1^*^		**Model 2** ^**†**^		**Model 3** ^**‡**^	
	**HR (95% CI)**	***p*-value**	**HR (95% CI)**	***p*-value**	**HR (95% CI)**	***p*-value**
Normal glucose regulation	-	0.317	-	0.180	-	0.319
Q2 vs. Q1	0.768 (0.537–1.099)	0.144	1.447 (0.982–2.132)	0.062	1.440 (0.942–2.201)	0.092
Q3 vs. Q1	1.018 (0.730–1.419)	0.069	1.406 (0.988–1.999)	0.058	1.403 (0.932–2.111)	0.105
Q4 vs. Q1	1.056 (0.791–1.411)	0.149	1.437 (1.001–2.065)	0.050	1.339 (0.897–1.998)	0.153
Prediabetes	-	0.714	-	0.535	-	0.767
Q2 vs. Q1	0.862 (0.541–1.374)	0.533	0.892 (0.556–1.431)	0.636	0.836 (0.510–1.370)	0.478
Q3 vs. Q1	1.074 (0.691–1.671)	0.750	1.184 (0.760–1.846)	0.455	1.046 (0.648–1.690)	0.853
Q4 vs. Q1	1.083 (0.702–1.671)	0.718	1.170 (0.758–1.806)	0.479	1.054 (0.654–1.700)	0.829
Diabetes	-	0.005	-	0.012	-	0.021
Q2 vs. Q1	0.916 (0.675–1.242)	0.572	0.999 (0.731–1.366)	0.996	0.938 (0.673–1.309)	0.707
Q3 vs. Q1	1.323 (1.008–1.736)	0.043	1.338 (1.010–1.773)	0.042	1.270 (0.925–1.742)	0.139
Q4 vs. Q1	1.409 (1.081–1.837)	0.011	1.446 (1.103–1.896)	0.008	1.445 (1.076–1.940)	0.014

* *Model 1 covariates: age, sex, and body mass index.*

† *Model 2 covariate: SS II.*

‡ *Model 3 covariates: SS II, treatment strategies (PCI, CABG, or MT), previous history of stroke, body mass index, blood pressure, LDL-C, hs-CRP, and liver enzymes*.

Meanwhile, the combination of DB with SS II resulted in significant improvements of discrimination and reclassification of SS II for all-cause death, cardiac death, or MACCE (Table 6). The improvement was more consistent in all-cause mortality

**Table 6:** Additional predictive value provided by DB beyond the SS II.

	**C-index (95% CI)**	***p*-value**	**NRI (95% CI)**	***p*-value**	**IDI (95% CI)**	***p*-value**
All-cause death
SS II	0.718 (0.711–0.736)	Reference	–	Reference	–	Reference
SS II + DB	0.724 (0.706–0.731)	0.013	0.151 (0.079–0.224)	<0.001	0.003 (0.001–0.004)	0.004
Cardiac death
SS II	0.713 (0.700–0.725)	Reference	–	Reference	–	Reference
SS II + DB	0.718 (0.705–0.730)	0.066	0.149 (0.054–0.2445)	0.002	0.002 (0.000–0.003)	0.063
MACCE
SS II	0.591 (0.578–0.605)	Reference	–	Reference	–	Reference
SS II + DB	0.594 (0.581–0.608)	0.155	0.072 (0.014–0.130)	0.015	0.001 (0.000–0.002)	0.034

*CI, confidence interval; IDI, integrated discrimination improvement; MACCE, major adverse cardiovascular and cerebrovascular events; NRI, net reclassification index; SS II, SYNTAX (Synergy between PCI with TAXUS and Cardiac Surgery) score II*.

## Discussion

This is a *post-hoc* analysis of a large, prospective cohort of 3VD patients in China. There are several findings about the effects of baseline DB in ACS patients with 3VD: (1) DB was positively associated with SS, which was not altered in multivariable analysis. (2) Heightened DB was an independent risk factor of all-cause death in the long term. (3) Subgroup analysis showed the positive relationship was significant in diabetic patients as well as those receiving conservative treatment. (4) Combination of DB significantly improved the discrimination and/or reclassification abilities of SS II for long-term outcomes.

In recent years, SS has been put forward as a preferable tool to evaluate the complexity and severity of coronary lesions and then to determine optimal strategy for 3VD or left main disease (3). The updated scoring system, SS II, which combines clinical risk factors with angiographical characteristics, further improves its predictive ability (19). Nevertheless, novel biomarkers are yet to be discovered to refine diagnosis and risk stratification.

Bilirubin is a member of the tetrapyrrole compound family. Heme oxygenase catalyzes heme to carbon monoxide, ferrous iron, and biliverdin. Biliverdin is subsequently metabolized to unconjugated bilirubin by biliverdin reductase. The latter is a non-polar molecule, and binding with plasma albumin, it is transported to the liver and then forms conjugates by UDP-glucuronosyltransferase (8).

Some research has reported that high-normal or mildly elevated bilirubin is a protector of cardiovascular disease. A typical example is that patients with Gilbert syndrome who have mild hyperbilirubinemia are less susceptible to cardiovascular disease (20). In view of the secondary prevention, Kuwano et al. reported that a low bilirubin level predicted a greater hazard of in-stent restenosis after PCI (21). A study following up ACS patients found significantly decreased 1-year cardiac death and major adverse cardiovascular events such as TB elevation (13). However, much less information is provided with the value of DB. Analyses derived from a large cohort of 14,583 participants suggested that DB was inversely associated with coronary artery calcium score (22). From basic experiments, the most convincing explanation is its antioxidative effects such as scavenging free radicals, inhibiting the formation of oxidized low-density lipoprotein (ox-LDL) (23, 24). Bilirubin also confers anti-inflammatory proprieties in its combat with inflammatory molecules (8). Furthermore, it is suggested that high physiological levels of bilirubin could inhibit the growth and proliferation of human coronary artery smooth muscle cells (25).

On the contrary, several recent studies argued that high bilirubin concentrations could portend poor condition of the ACS population. Celik et al. demonstrated that a high TB level was independently associated with no reflow as well as increased in-hospital MACCE in patients with STEMI undergoing primary PCI (26). Sahin et al. reported that TB was an independent risk factor for SS in patients with STEMI (odds ratio 1.86, 95% CI 1.04–3.35, *p* = 0.038) (27). A similar correlation between TB and SS was also shown in another research of NSTEMI (28). When it extends to all ACS patients, supportive evidence from a cohort study of coronary artery disease showed that TB > 0.60 mg/dl significantly predicted worse 3-year outcomes. And this effect was most attributable to ACS subgroups (14). In light of DB, Xu et al. evaluated TB together with its subtypes in ACS and pointed out that high DB was an independent predictor for long-term outcomes (29). Notably, DB was superior to TB in predictive capacity, while indirect bilirubin was of no significance.

Different from previous work that 3VD or diabetes accounted for a minority, our study is the first to focus on the association between DB and the high-risk ACS patients with confirmed three-vessel stenosis. Besides, unlike those mostly including patients with PCI, surgical and conservative therapy added up to over half of all the cases in the present analysis. From multiple analyses, we demonstrated distinct perils of DB in this cohort, while TB failed to be an independent variable. The result can be explained from several aspects. Firstly, bilirubin serves certainly as a potent antioxidant. However, induction of bilirubin following the stress may not counteract the damage of oxidative substances, making its protective effect not significant. Previous studies observed its upregulation in the setting of STEMI. Therefore, it is reasonable that elevated DB is a sensitive marker of intense pathological oxidative process in an acute ischemic attack. Secondly, the parallel correlations of DB with C-reactive protein and white blood cell might mirror the systemic inflammatory status at the root of ACS. A cohort study of STEMI showed that the combined effect of a higher white cell count and TB was better than a single biomarker in prediction of in-hospital complications and in-hospital mortality (30). As for its significant predictivity for long-term outcomes in our study, stenosis of three major coronary arteries implied a lasting and advancing activity of atherosclerosis. Combining the fact that DB was only notable in the conservative therapy subgroup, our observations offer a clue that the aforementioned biological interactions actively lie in untreated lesions. Similar explanations could also be applied to diabetes. Diabetic patients are often characterized by more complex, extensive, and diffuse coronary lesions. As introduced in the beginning, abnormal bilirubin levels were observed in the diabetic patients (16, 17). Together with our studies, DB was a surrogate biomarker of oxidative and inflammatory responses, the common mechanisms in both ACS and diabetes. Even though major lesions were treated, the effect of DB lasted because of the nature of chronic inflammation status and extensive lesions. Evidently, the protective effect of DB could not eliminate the pathological process. In addition, adjusting other confounding biomarkers did not attenuate its predictivity, suggesting that the panoramic image of two diseases await further exploration.

It is unclear why DB not TB emerges as an independent predictor. Given the disparities in ejection fraction (EF) and NT-proBNP across DB quartiles, a plausible line is that a higher DB might be a sign of cardiac dysfunction leading to insufficient perfusion and/or venous congestion and, consequently, impaired liver injury (31, 32). A cohort study followed up 556 patients with acute decompensated heart failure (HF) for 338 ± 252 days. They found that baseline DB was an independent predictor of adverse events (adjusted HR 1.052, 95% CI 1.001–1.099, *p* = 0.034), while TB was not (33). As for venous congestion, although our database has little relative information on right HF, previous studies demonstrated that hyperbilirubinemia was associated with advanced HF (34, 35). Thus, it is reasonable to assume that elevated DB paralleled unstable hepatic hemodynamics, which was probably not well-manifested by traditional measures (EF and NT-proBNP). Still, this is debatable as there is limited knowledge about this topic at present.

## Limitations

There are some limits in the present study. Firstly, this study cannot conclude causal relationships due to the nature of observational research. Secondly, the present findings should be interpreted with caution because of the lack of detailed information on hepatobiliary impairments or other related diseases. Similarly, although multivariable regression analyses were conducted, some unknown factors might bias the results. Thirdly, it is uncertain whether fluctuation of DB during the long follow-up period has a confounding effect on the outcomes.

In conclusion, findings on this high-risk population demonstrate that baseline DB is an independent risk factor for the complexity of lesions and long-term prognosis. The present observations shed light on the potential of DB in risk stratification.

## Data Availability Statement

The datasets presented in this article are not readily available because There are regulations from our scientific research department that we researchers shall not provide any data for others. Requests to access the datasets should be directed to ljxufuwai@126.com.

## Ethics Statement

The studies involving human participants were reviewed and approved by the Review Board of Fuwai Hospital. The patients/participants provided their written informed consent to participate in this study.

## Author Contributions

LJ, JT, X-yZ, J-jX, RL, and L-jX contributed to the data acquisition of the work. R-tH, R-lG, J-qY, and LS contributed to the conceptualization, project administration, and supervision. YL and CZ analyzed and drafted the manuscript. L-jX and LS critically revised the manuscript. All authors contributed to the article and approved the submitted version.

## Conflict of Interest

The authors declare that the research was conducted in the absence of any commercial or financial relationships that could be construed as a potential conflict of interest.

## Publisher's Note

All claims expressed in this article are solely those of the authors and do not necessarily represent those of their affiliated organizations, or those of the publisher, the editors and the reviewers. Any product that may be evaluated in this article, or claim that may be made by its manufacturer, is not guaranteed or endorsed by the publisher.
